# Ripretinib versus sunitinib in gastrointestinal stromal tumor: ctDNA biomarker analysis of the phase 3 INTRIGUE trial

**DOI:** 10.1038/s41591-023-02734-5

**Published:** 2024-01-05

**Authors:** Michael C. Heinrich, Robin L. Jones, Suzanne George, Hans Gelderblom, Patrick Schöffski, Margaret von Mehren, John R. Zalcberg, Yoon-Koo Kang, Albiruni Abdul Razak, Jonathan Trent, Steven Attia, Axel Le Cesne, Brittany L. Siontis, David Goldstein, Kjetil Boye, Cesar Sanchez, Neeltje Steeghs, Piotr Rutkowski, Mihaela Druta, César Serrano, Neeta Somaiah, Ping Chi, William Reichmann, Kam Sprott, Haroun Achour, Matthew L. Sherman, Rodrigo Ruiz-Soto, Jean-Yves Blay, Sebastian Bauer

**Affiliations:** 1 Division of Hematology/Oncology, Portland VA Health Care System, Portland, OR USA; 2 Department of Medicine, OHSU Knight Cancer Institute, Portland, OR USA; 3 Sarcoma Unit, The Royal Marsden NHS Foundation Trust and Institute of Cancer Research, London, UK; 4 Center for Sarcoma and Bone Oncology, Dana-Farber Cancer Institute, Boston, MA USA; 5 Department of Medical Oncology, Leiden University Medical Center, Leiden, Netherlands; 6 Department of General Medical Oncology, University Hospitals Leuven, Leuven Cancer Institute, KU Leuven, Leuven, Belgium; 7 Department of Hematology/Oncology, Fox Chase Cancer Center, Temple University Health System, Philadelphia, PA USA; 8 Department of Medical Oncology, Monash University School of Public Health and Preventive Medicine, Alfred Health, Melbourne, Victoria Australia; 9 Department of Oncology, Asan Medical Center, University of Ulsan, Seoul, Korea; 10 Division of Medical Oncology, Toronto Sarcoma Program, Princess Margaret Cancer Center, Toronto, ON Canada; 11 Department of Medical Oncology, Sylvester Comprehensive Cancer Center, University of Miami Health System, Miami, FL USA; 12 Department of Medical Oncology, Mayo Clinic, Jacksonville, FL USA; 13 Medical Oncology Department, Gustave Roussy, Villejuif, France; 14 Department of Medical Oncology, Mayo Clinic, Rochester, MN USA; 15 Department of Medical Oncology, Prince of Wales Hospital, Randwick, New South Wales Australia; 16 Department of Tumor Biology, Oslo University Hospital, Oslo, Norway; 17 Department of Hematology-Oncology, Centro de Cáncer, Hospital Clínico Pontificia Universidad Católica de Chile, Santiago, Chile; 18 Department of Medical Oncology, The Netherlands Cancer Institute, Antoni van Leeuwenhoek Hospital, Amsterdam, Netherlands; 19 Department of Soft Tissue/Bone Sarcoma and Melanoma, Maria Sklodowska-Curie National Research Institute of Oncology, Warszawa, Poland; 20 Sarcoma Program, Moffitt Cancer Center, Tampa, FL USA; 21 Sarcoma Translational Research Group, Vall d’Hebron Institute of Oncology, Barcelona, Spain; 22 Department of Sarcoma Medical Oncology, The University of Texas MD Anderson Cancer Center, Houston, TX USA; 23 Department of Medicine, Memorial Sloan Kettering Cancer Center, New York, NY USA; 24 Department of Medicine, Weill Cornell Medicine, New York, NY USA; 25 Biometrics, Deciphera Pharmaceuticals, LLC, Waltham, MA USA; 26 Translational Medicine, Deciphera Pharmaceuticals, LLC, Waltham, MA USA; 27 Clinical Development, Deciphera Pharmaceuticals, LLC, Waltham, MA USA; 28 Department of Medical Oncology, Centre Léon Bérard, Lyon, France; 29 Department of Medical Oncology and Sarcoma Center, West German Cancer Center, University Hospital Essen, University Duisburg-Essen, Essen, Germany; 30 German Cancer Consortium (DKTK), Partner Site University Hospital Essen, Essen, Germany

**Keywords:** Sarcoma, Cancer therapeutic resistance, Targeted therapies, Oncogenes, Cancer genomics

## Abstract

INTRIGUE was an open-label, phase 3 study in adult patients with advanced gastrointestinal stromal tumor who had disease progression on or intolerance to imatinib and who were randomized to once-daily ripretinib 150 mg or sunitinib 50 mg. In the primary analysis, progression-free survival (PFS) with ripretinib was not superior to sunitinib. In clinical and nonclinical studies, ripretinib and sunitinib have demonstrated differential activity based on the exon location of *KIT* mutations. Therefore, we hypothesized that mutational analysis using circulating tumor DNA (ctDNA) might provide further insight. In this exploratory analysis (*N* = 362), baseline peripheral whole blood was analyzed by a 74-gene ctDNA next-generation sequencing–based assay. ctDNA was detected in 280/362 (77%) samples with *KIT* mutations in 213/362 patients (59%). Imatinib-resistant mutations were found in the KIT ATP-binding pocket (exons 13/14) and activation loop (exons 17/18). Mutational subgroup assessment showed 2 mutually exclusive populations with differential treatment effects. Patients with only *KIT* exon 11 + 13/14 mutations (ripretinib, *n* = 21; sunitinib, *n* = 20) had better PFS with sunitinib versus ripretinib (median, 15.0 versus 4.0 months). Patients with only *KIT* exon 11 + 17/18 mutations (ripretinib, *n* = 27; sunitinib, *n* = 25) had better PFS with ripretinib versus sunitinib (median, 14.2 versus 1.5 months). The results of this exploratory analysis suggest ctDNA sequencing may improve the prediction of the efficacy of single-drug therapies and support further evaluation of ripretinib in patients with *KIT* exon 11 + 17/18 mutations. ClinicalTrials.gov identifier: NCT03673501.

## Abstract (web summary)

Exploratory ctDNA analyses from the phase 3 INTRIGUE trial indicate that ripretinib may provide benefits in patients with advanced gastrointestinal stromal tumors with *KIT* exon 11 + 17/18 mutations

## Main

Gastrointestinal stromal tumor (GIST) is the most common gastrointestinal sarcoma, with approximately 80% of cases driven by mutations in *KIT*, and up to 10% by mutations in platelet-derived growth factor receptor α (*PDGFRA*)^1–3^. Imatinib, a KIT/PDGFRA tyrosine kinase inhibitor (TKI), is an effective first-line therapy for patients with advanced GIST; however, most patients ultimately develop disease progression due to secondary resistance mutations^4–10^. Approximately 90% of patients with *KIT*-mutant GIST who had disease progression on imatinib harbor newly acquired secondary *KIT* mutations, which most commonly appear in the ATP-binding pocket (encoded by exons 13/14) and/or activation loop (exons 17/18)^11–15^.

Sunitinib is the approved second-line therapy for patients with advanced GIST following progression on or intolerance to imatinib^16^. In the registrational phase 3 trial, patients treated with sunitinib demonstrated an overall median progression-free survival (PFS) of 5.6 months. However, there was no analysis of secondary mutations in the phase 3 trial, and sunitinib has demonstrated differential efficacy dependent on the location of imatinib-resistant *KIT* mutations^11,17^. In a phase 1/2 study (NCT00457743), the median PFS for sunitinib was 7.8 months in patients harboring secondary resistance mutations in the KIT ATP-binding pocket compared with 2.3 months in patients who had mutations in the activation loop^11^.

Ripretinib, a switch-control TKI, is approved for adult patients with advanced GIST who have received prior treatment with three or more TKIs, including imatinib, based on the results of the phase 3 INVICTUS study^18,19^. When compared with sunitinib in the phase 3 INTRIGUE trial, ripretinib demonstrated similar efficacy in patients who had disease progression on or were intolerant to imatinib in the *KIT* exon 11 intent-to-treat (ITT; median PFS, 8.3 versus 7.0 months, respectively; *P* = 0.36) and overall ITT populations (median PFS, 8.0 versus 8.3 months, respectively; nominal *P* = 0.72), suggesting that ripretinib demonstrated comparable efficacy to sunitinib as a second-line therapy^20^. Ripretinib also demonstrated a more favorable safety profile compared with sunitinib, with fewer patients experiencing grade 3/4 treatment-emergent adverse events (TEAEs)^20^. Based on these primary results from the INTRIGUE trial, ripretinib was recently included in the National Comprehensive Cancer Network Clinical Practice Guidelines in Oncology for GIST (version 1.2023) as a preferred second-line regimen for patients with advanced GIST who are intolerant to sunitinib^20,21^. As fourth-line or later therapy, PFS with ripretinib was longer than placebo in all assessed mutational subgroups (*KIT* exons 9, 11, 13 and 17), suggesting broad activity in this later-line setting, irrespective of baseline mutation status^22^.

Tumor tissue obtained through biopsy has been the predominant source for mutational analysis in cancer; however, circulating tumor DNA (ctDNA) analysis is becoming more common^23,24^. Despite the limitations of using ctDNA (e.g., sample processing issues, assay specificity, low shedding disease), these analyses may provide more comprehensive information reflective of systemic tumor burden rather than the limited areas sampled by tissue biopsy^24–26^. Given the differential activity of TKIs depending on the location of *KIT* mutations as well as the poor activity of sunitinib in patients with secondary *KIT* exon 17/18 mutations, we hypothesized that further investigation by mutational subgroup using ctDNA could provide more insight into the efficacy of these agents as second-line therapies. In this prespecified exploratory analysis from INTRIGUE, we present the landscape of *KIT* mutations at the onset of imatinib failure and evaluate the efficacy of ripretinib versus sunitinib in patients with advanced GIST according to baseline *KIT* mutation status as determined by ctDNA analysis.

## Results

### ctDNA sample evaluability and landscape of *KIT* mutations

**Fig. 1:**
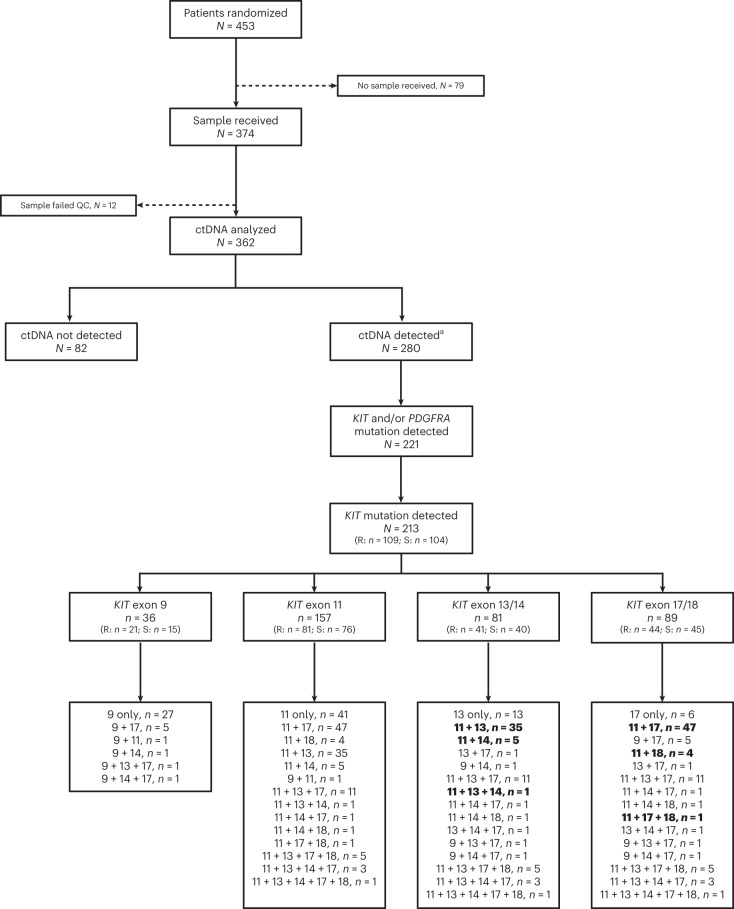
ctDNA analysis and detection. Patients are included in multiple groups if they had more than one mutation; patients can have multiple mutations in the same exon. Groups under each of the categories (*KIT* exon 9, 11, 13/14 or 17/18) are not mutually exclusive, and patients may appear in more than one box. Bold indicates patients who were included in the analysis populations for the current manuscript. CNV, copy number variation; QC, quality control; R, ripretinib; S, sunitinib. ^**a**^ctDNA detected only for SNV/INDEL; two patients had CNV-only mutations and were categorized as ctDNA not detected.

**Extended Data Fig. 1:**
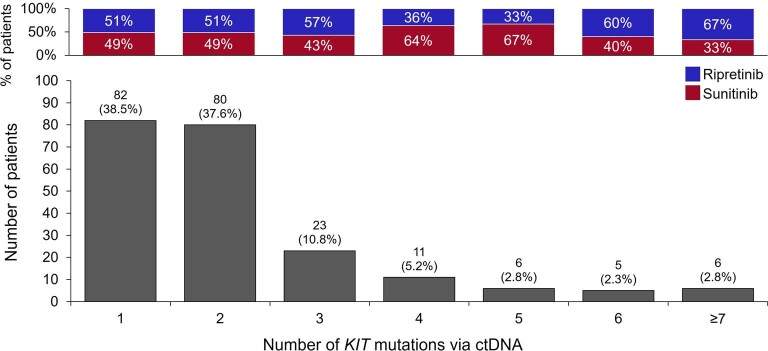
Frequency of *KIT* mutations. Includes both primary and secondary mutations. Only somatic *KIT* mutations considered; sunitinib arm includes 2 patients not treated. Total patients, N = 213. ctDNA, circulating tumor DNA.

**Extended Data Fig. 2:**
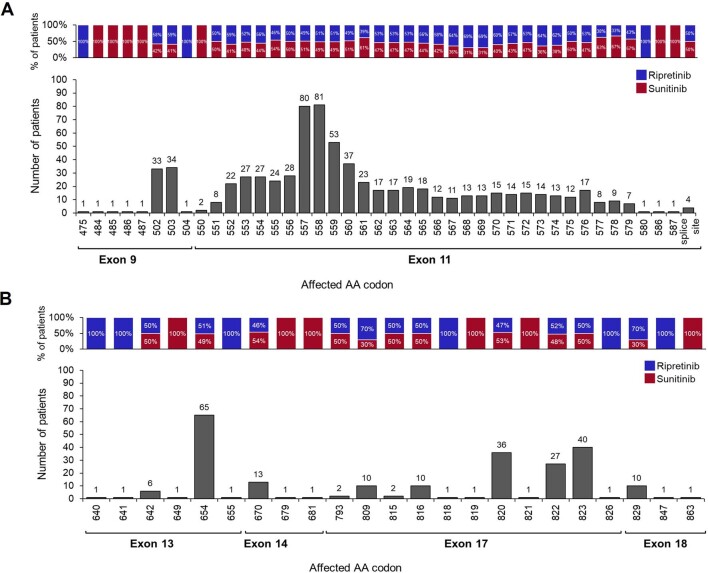
Number of patients with *KIT* primary (A) and secondary resistance (B) mutations. Data indicate number of patients with mutations at all affected codons; each patient can have multiple mutations. AA, amino acid.

Of 453 patients in the overall ITT population, ctDNA was analyzed for 362 (80%; Fig. 1). A total of 280/362 patients (77%) had detectable ctDNA for single-nucleotide variants (SNVs) and/or insertions/deletions (INDELs); 213/362 (59%) had detectable *KIT* mutations (Fig. 1). The observed *KIT* mutations included *KIT* exon 9 (*n* = 36/213 (16.9%)), exon 11 (*n* = 157/213 (73.7%)), exons 13/14 (*n* = 81/213 (38.0%)) and exons 17/18 (*n* = 89/213 (41.8%)), with patients belonging to more than one group if they harbored multiple mutations (Fig. 1). Most patients harbored one or two *KIT* mutations (162 (76%)), with 6 patients exhibiting seven or more *KIT* mutations (Extended Data Fig. 1). Primary *KIT* exon 9 mutations were detected in 36 patients, with the most common being the AY duplication at codons 502–503 (*n* = 33 mutations); 157 patients had *KIT* exon 11 primary mutations, with codons 557–558 being the most impacted (*n* = 82 mutations; Extended Data Fig. 2a).

**Fig. 2:**
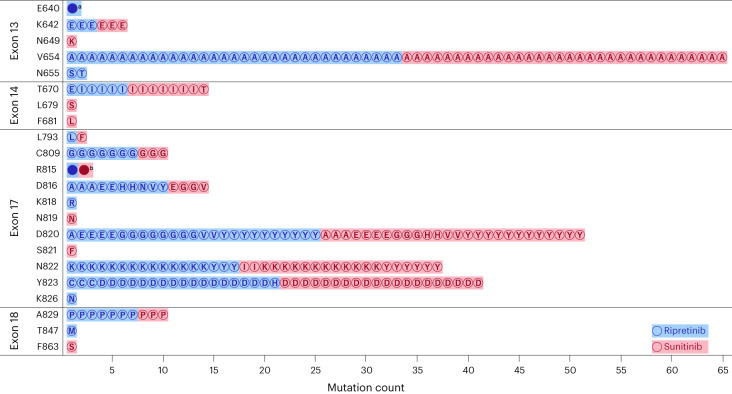
Heterogeneity of ctDNA mutations in the KIT ATP-binding pocket (exons 13/14) and activation loop (exons 17/18). This plot illustrates the number of mutations; each patient could have multiple mutations. The letters in the bubbles and in front of each listed codon represent amino acids. A, alanine; ATP, adenosine triphosphate; C, cysteine; D, aspartic acid; E, glutamic acid; F, phenylalanine; G, glycine; H, histidine; I, isoleucine; K, lysine; L, leucine; M, methionine; N, asparagine; P, proline; R, arginine; S, serine; T, threonine; V, valine; Y, tyrosine. ^**a**^E640_L641delinsD. ^**b**^Ripretinib: R815_D816delinsN; sunitinib: R815_D816delinsK.

Most patients had secondary resistance mutations in *KIT* exon 13 and/or exon 17 (Extended Data Fig. 2b). Overall, 42 unique secondary resistance mutations were observed in the KIT ATP-binding pocket (exons 13/14) and activation loop (exons 17/18; Fig. 2). The most common secondary resistance mutation was the V654A substitution in exon 13 (*n* = 65) followed by the Y823D (*n* = 37) and N822K (*n* = 26) substitutions in exon 17 (Fig. 2).

**Fig. 3:**
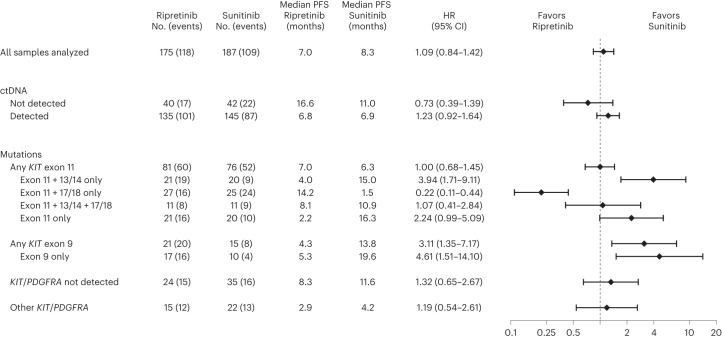
Forest plot of PFS by mutational subgroup. Data are represented as hazard ratio (HR) ± 95% confidence interval (CI). PFS was summarized using the Kaplan-Meier method with associated two-sided 95% CIs calculated using the Brookmeyer and Crowley method. HRs were obtained from the unstratified Cox proportional hazard model. Nine patients were included in multiple groups, including one patient with mutations in *KIT* exon 9 and *KIT* exon 11 and eight patients with mutations in *KIT* exon 11 and *PDGFRA*; the exon 11 + 13/14-only group excludes patients with mutations in *KIT* exons 9, 17 and 18; the exon 11 + 17/18-only group excludes patients with mutations in *KIT* exons 9, 13 and 14. Data cutoff: 1 September 2021.

When looking at median PFS by mutation subgroup, two diametrically opposed populations were evident (Fig. 3). Differential treatment effects were observed in patients with primary *KIT* exon 11 mutations with imatinib-resistant mutations exclusively in exons 13/14 (41/362 (11%)) and in patients with primary *KIT* exon 11 mutations with imatinib-resistant mutations exclusively in exons 17/18 (52/362 (14%)). Based on these findings, outcome results for these two mutually exclusive, diametrically opposed populations will be presented in the current article.

### Patients

Demographics and clinical characteristics for the overall ITT population were published previously^20^. Baseline demographic and clinical characteristics were well balanced between the *KIT* exon 11 + 13/14 and *KIT* exon 11 + 17/18 populations and between treatment arms (Table 1). The median age was 59.0 and 60.0 years in the *KIT* exon 11 + 13/14 and *KIT* exon 11 + 17/18 populations, respectively. Race was self-reported, and most patients were White males from North America or Europe (Table 1).

**Table 1:** **Patient demographics and baseline characteristics in the** ***KIT*** **exon 11** + **13/14 and** ***KIT*** **exon 11** **+** **17/18 populations**

Characteristic	*KIT* exon 11 + 13/14			*KIT* exon 11 + 17/18		
	Ripretinib	Sunitinib	Total	Ripretinib	Sunitinib	Total
	*n* = 21	*n* = 20	*N* = 41	*n* = 27	*n* = 25	*N* = 52
**Age (years), median (min, max)**	57.0 (34, 79)	61.5 (43, 74)	59.0 (34, 79)	59.0 (33, 80)	63.0 (31, 88)	60.0 (31, 88)
**Sex (male), no. (%)**	12 (57.1)	9 (45.0)	21 (51.2)	18 (66.7)	20 (80.0)	38 (73.1)
**Race (White), no. (%)**	15 (71.4)	12 (60.0)	27 (65.9)	17 (63.0)	20 (80.0)	37 (71.2)
**Region, no. (%)**						
North America	12 (57.1)	8 (40.0)	20 (48.8)	12 (44.4)	9 (36.0)	21 (40.4)
South America	1 (4.8)	1 (5.0)	2 (4.9)	0	0	0
Europe	8 (38.1)	9 (45.0)	17 (41.5)	12 (44.4)	14 (56.0)	26 (50.0)
Asia-Pacific	0	2 (10.0)	2 (4.9)	3 (11.1)	2 (8.0)	5 (9.6)
**Primary tumor site, no. (%)**						
Gastric	12 (57.1)	9 (45.0)	21 (51.2)	9 (33.3)	13 (52.0)	22 (42.3)
Nongastric	9 (42.9)	11 (55.0)	20 (48.8)	18 (66.7)	12 (48.0)	30 (57.7)
**ECOG PS, no. (%)**						
0	13 (61.9)	13 (65.0)	26 (63.4)	18 (66.7)	9 (36.0)	27 (51.9)
1	7 (33.3)	7 (35.0)	14 (34.1)	9 (33.3)	15 (60.0)	24 (46.2)
2	1 (4.8)	0	1 (2.4)	0	1 (4.0)	1 (1.9)
**Imatinib intolerance, no. (%)**	2 (9.5)	0	2 (4.9)	0	1 (4.0)	1 (1.9)
**Sum of longest diameters of target lesions (mm), median (min, max)**	106.8 (47, 373)	108.3 (15, 418)	106.8 (15, 418)	119.9 (33, 392)	124.8 (24, 368)	120.8 (24, 392)
**Duration of imatinib therapy (months), median (min, max)**	26.87 (7.8, 119.0)	55.80 (18.9, 186.2)	46.75 (7.8, 186.2)	55.66 (11.9, 203.4)	56.54 (3.3, 190.1)	56.10 (3.3, 203.4)

ECOG PS, Eastern Cooperative Oncology Group Performance Status; max, maximum; min, minimum.

### Efficacy

**Fig. 4:**
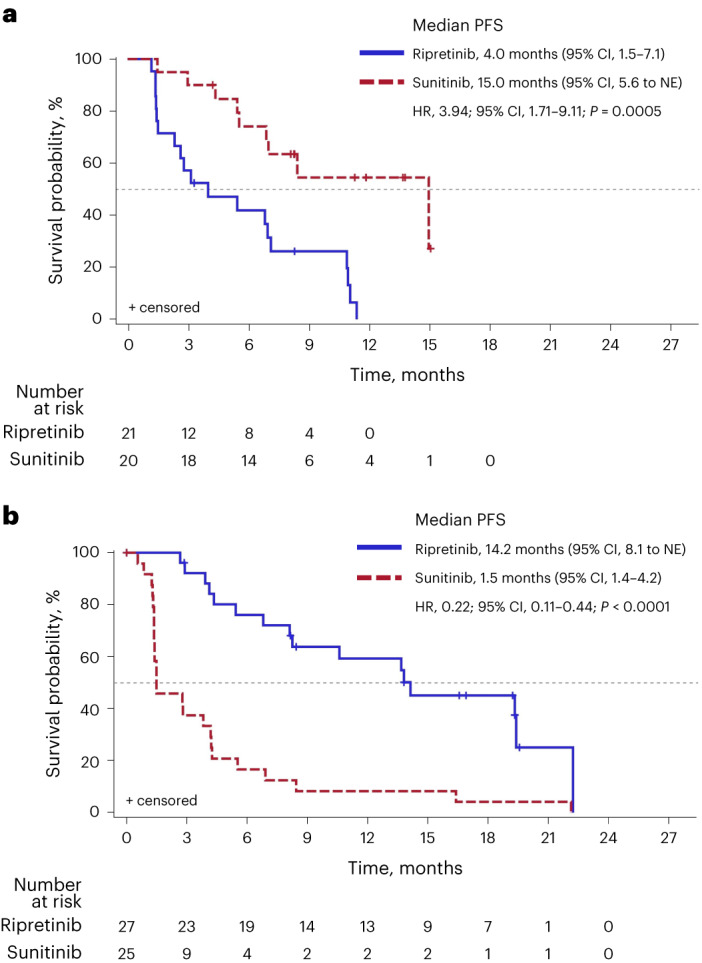
Kaplan-Meier analysis of PFS for patients treated with ripretinib or sunitinib in the *KIT* exon 11 + 13/14 (**a**) and *KIT* exon 11 + 17/18 (**b**) populations. PFS was summarized using the Kaplan-Meier method with associated two-sided 95% CIs calculated using the Brookmeyer and Crowley method. HRs and *P* values were obtained from the unstratified Cox proportional hazard model and two-sided unstratified log-rank tests, respectively. Data cutoff: 1 September 2021. *P* values are nominal. NE, not estimable.

In the *KIT* exon 11 + 13/14 population, sunitinib demonstrated improved PFS compared with ripretinib (median, 15.0 versus 4.0 months; HR, 3.94; 95% CI, 1.71–9.11; nominal *P* = 0.0005; Figs. 3 and 4a). Conversely, ripretinib demonstrated improved PFS compared with sunitinib in patients with *KIT* exon 11 + 17/18 mutations (median, 14.2 versus 1.5 months; HR, 0.22; 95% CI, 0.11–0.44; nominal *P* < 0.0001; Figs. 3 and 4b). These results remained robust when accounting for multiple treatment comparisons across mutational subgroups, with a significant interaction between treatment and mutational subgroup (nominal *P* < 0.0001; Extended Data Table 1).

Similar to the PFS for the overall ITT and *KIT* exon 11 ITT populations in the primary analysis, PFS rates for the total (all samples analyzed) and any *KIT* exon 11 groups were similar between treatment arms (Fig. 3)^20^. PFS was better with sunitinib versus ripretinib for patients with only baseline *KIT* exon 11 mutations (median, 16.3 versus 2.2 months; HR, 2.24; 95% CI, 0.99–5.09; nominal *P* = 0.0460; Fig. 3). In patients with co-occurring imatinib-resistant secondary mutations in both the KIT ATP-binding pocket and activation loop (*n* = 22/362 (6%)), no difference was revealed between sunitinib and ripretinib (HR, 1.07; 95% CI, 0.41–2.84; nominal *P* = 0.8843; Fig. 3).

**Fig. 5:**
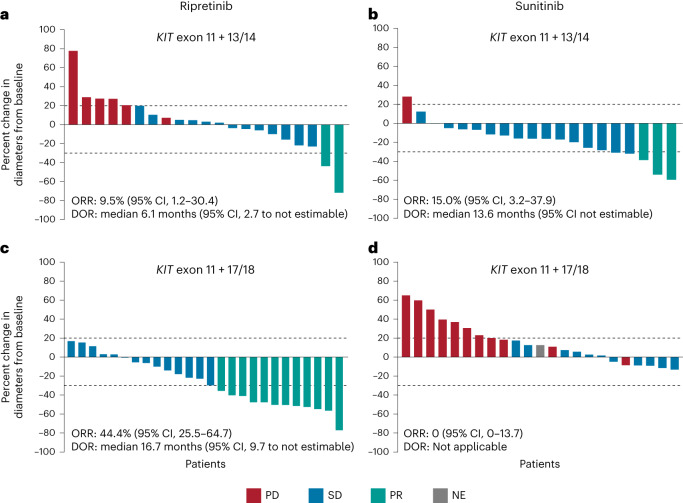
Best percent change from baseline in the sum of target lesion diameters in patients treated with ripretinib or sunitinib. **a**,**b**, Patients with *KIT* exon 11 + 13/14 mutations. **c**,**d**, Patients with *KIT* exon 11 + 17/18 mutations. Data cutoff: 1 September 2021. Dotted line at 20% represents the threshold for PD; dotted line at −30% represents threshold for PR. DOR, duration of response; NE, not evaluable; PD, progressive disease; PR, partial response; SD, stable disease.

**Extended Data Fig. 3:**
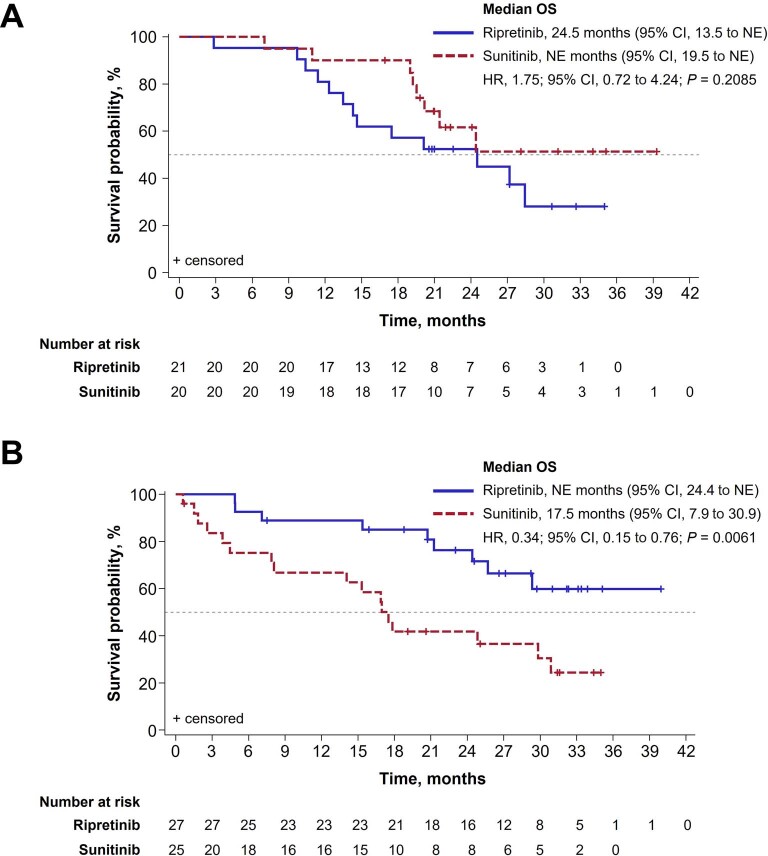
Kaplan-Meier analysis of OS for patients treated with ripretinib or sunitinib in the *KIT* exon 11 + 13/14 (A) and *KIT* exon 11 + 17/18 (B) populations. OS was summarized using the Kaplan-Meier method with associated 2-sided 95% CIs calculated using the Brookmeyer and Crowley method. HRs and *P*-values were obtained from the unstratified Cox proportional hazard model and 2-sided unstratified log-rank tests, respectively. Data cutoff: 1 September 2022. *P*-values are nominal. CI, confidence interval; HR, hazard ratio; NE, not estimable; OS, overall survival.

In the *KIT* exon 11 + 13/14 population, the objective response rate (ORR) was 9.5% with ripretinib versus 15.0% with sunitinib (response difference (RD), −5.5%; 95% CI, −27.6 to 16.2; nominal *P* = 0.5922; Fig. 5a,b). A higher ORR was observed with ripretinib versus sunitinib in the *KIT* exon 11 + 17/18 population (44.4% versus 0%; RD, 44.4%; 95% CI, 23.0–62.7; nominal *P* = 0.0001; Fig. 5c,d). Across both treatment arms, overall survival (OS) event rates were 51.2% and 50.0% in the *KIT* exon 11 + 13/14 and *KIT* exon 11 + 17/18 populations, respectively. Median OS was not reached for patients receiving sunitinib in the *KIT* exon 11 + 13/14 population, whereas the median OS for patients receiving ripretinib was 24.5 months (HR, 1.75; 95% CI, 0.72–4.24; nominal *P* = 0.2085; Extended Data Fig. 3a) with a median follow-up of 24.1 and 30.7 months for sunitinib and ripretinib, respectively. Improved OS was observed with ripretinib versus sunitinib in the *KIT* exon 11 + 17/18 population (median, not reached versus 17.5 months; HR, 0.34; 95% CI, 0.15 to 0.76; nominal *P* = 0.0061; Extended Data Fig. 3b) with a median follow-up of 29.7 months for ripretinib and 31.4 months for sunitinib. These results remained robust when accounting for multiple treatment comparisons across mutational subgroups, with a significant interaction between treatment and mutational subgroup (nominal *P* = 0.0179; Extended Data Table 2).

### Safety and follow-up therapies

Median treatment duration for ripretinib versus sunitinib in the *KIT* exon 11 + 13/14 population was 4.6 versus 9.5 months, respectively. In the *KIT* exon 11 + 17/18 population, median treatment duration was 14.0 versus 3.0 months for patients receiving ripretinib versus sunitinib, respectively (Extended Data Table 3). The observed safety profile appeared to be consistent with the primary analysis. There were more grade 3/4 drug-related TEAEs in patients receiving sunitinib versus ripretinib (*KIT* exon 11 + 13/14: 50.0% versus 28.6%; *KIT* exon 11 + 17/18: 50.0% versus 33.3%, respectively; Extended Data Table 3). In the *KIT* exon 11 + 13/14 population, more patients receiving sunitinib versus ripretinib underwent dose interruptions and reductions due to any TEAE (30.0% versus 23.8% and 45.0% versus 4.8%, respectively; Extended Data Table 3). Conversely, in the *KIT* exon 11 + 17/18 population, more patients receiving ripretinib versus sunitinib underwent dose interruptions and dose reductions due to any TEAE (59.3% versus 41.7% and 37.0% versus 29.2%, respectively). However, when looking at the number of dose interruptions and reductions due to any TEAE in the first 12 weeks of the study, the proportions were either higher with sunitinib compared with ripretinib or comparable between the 2 arms (Extended Data Table 3). The most common TEAE of any grade observed with ripretinib was alopecia, regardless of mutational subgroup (*KIT* exon 11 + 13/14: 66.7%; *KIT* exon 11 + 17/18: 77.8%); the most common TEAEs with sunitinib in the *KIT* exon 11 + 13/14 and 11 + 17/18 populations were palmar-plantar erythrodysesthesia syndrome (60.0%) and hypertension (50.0%), respectively (Extended Data Table 4). Anticancer therapies received following discontinuation of study treatment can be found in Extended Data Table 5.

## Discussion

This exploratory analysis from the phase 3 INTRIGUE trial in pretreated, advanced GIST demonstrates the potential value of ctDNA next-generation sequencing (NGS)-based analysis of imatinib-resistant secondary *KIT* mutations to select second-line treatment. In this analysis, there were 42 unique mutations in the KIT ATP-binding pocket (exons 13/14) and activation loop (exons 17/18). The vast majority of these variants are known to cause imatinib resistance, but some of the novel variants with uncertain significance may not. In patients with the most common class of primary driver mutation in GIST (*KIT* exon 11 mutation), imatinib-resistant secondary mutations in the KIT ATP-binding pocket correlated with clinical benefit from sunitinib versus ripretinib (median PFS, 15.0 versus 4.0 months, respectively; *P* = 0.0005), whereas secondary mutations in the KIT activation loop indicated clinical benefit from ripretinib but not sunitinib (median PFS, 14.2 versus 1.5 months, respectively; *P* < 0.0001). Although these results are limited by the exploratory nature of the analysis, the differences in these populations were robust when accounting for multiple treatment comparisons across mutational subgroups with or without adjustment for different baseline characteristics.

Although mutational testing is strongly recommended for optimal therapy of patients with treatment-naïve, advanced GIST before initiating therapy with TKIs^21^, it is only performed in a minority of patients in the United States^27^. The primary genotype determines selection of drug (and dose) for imatinib (*KIT* exon 11 versus *KIT* exon 9), as well as avapritinib (*PDGFRA* exon 18 D842V mutation)^1,28,29^ and NTRK and BRAF inhibitors for patients with activating mutations in these kinases^30–32^. However, other than baseline mutation testing, there are limited studies supporting routine analysis of secondary mutations to optimize the treatment decision for the next line of therapy^15^. At most GIST centers, patients with *KIT*-mutant GIST are treated sequentially with imatinib, sunitinib, regorafenib and ripretinib, as first- to fourth-line therapies, based on progression or intolerance during the previous line of the therapy^21^. In the primary results from the INTRIGUE study, ripretinib was not superior to sunitinib as a second-line therapy in terms of PFS in a molecularly unselected population^20^. The current exploratory analysis, however, suggests that ctDNA could identify a molecular subset of patients who may preferentially benefit from second-line treatment with ripretinib rather than with the recommended second-line therapy, sunitinib^21^.

To date, mutational analysis has been predominantly performed on tissue biopsy samples; however, tissue biopsies are an invasive procedure that sample a portion of a single-tumor lesion, and multiple biopsies within and/or across lesions are not justifiable in routine clinical practice^23^. Plasma ctDNA analysis can theoretically overcome these limitations, with easy access to blood and the potential to reflect the full mutational burden across multiple metastatic sites and identify patients who might benefit from specific cancer therapies^25,26^. Some reports indicate low ctDNA shedding in GIST; however, higher rates of ctDNA detection were observed in active, metastatic disease^15,33,34^, and the current study demonstrated a high rate of ctDNA detection in patients with advanced GIST previously treated with imatinib (280/362; 77%). A previous study demonstrated good concordance between ctDNA and NGS testing from tumor tissue in a small cohort of patients with metastatic GIST^15^. Furthermore, imatinib-resistant mutations have been detected in ctDNA samples that were not observed in tissue biopsies, suggesting that ctDNA assays may allow physicians and researchers to effectively monitor secondary resistance mutations and treatment in advanced GIST^22,35,36^. To this end, these data may support a ctDNA-guided treatment approach in GIST using a sensitive and minimally invasive test and require further investigation in prospective trials.

The differential activity of sunitinib against imatinib-resistant mutations in the KIT ATP-binding pocket and activation loop was previously documented in both clinical and nonclinical studies^11,37,38^. In a nonrandomized, single-arm trial evaluating sunitinib in patients with advanced GIST, imatinib-resistant secondary mutations within the KIT activation loop (detected in single-tumor biopsies) were associated with rapid clinical progression (median PFS, 2.3 months), whereas PFS was significantly longer for patients with secondary mutations in the KIT ATP-binding pocket (median PFS, 7.8 months; *P* = 0.0157)^11^. We hypothesized that ctDNA could be particularly helpful in determining effective single-drug treatment approaches as it appears there is a subset of patients with advanced GIST who may not benefit from second-line treatment with sunitinib.

Preclinically, ripretinib inhibited a broad panel of *KIT* mutants in GIST and non-GIST cell lines, including many of the common primary and secondary resistance mutations observed in patients with advanced GIST^38^. Ripretinib was less effective, however, against secondary mutations in the KIT ATP-binding pocket than in the activation loop, regardless of primary mutation (*KIT* exon 11 or 9). In contrast, ripretinib demonstrated clinical activity compared with placebo independent of baseline mutation status in patients with fourth-line advanced GIST, including in a subgroup harboring KIT ATP-binding pocket mutations (*KIT* exon 13)^22^. However, this subgroup included any patient with a *KIT* exon 13 mutation regardless of additional activation loop mutations, and the study did not have an active comparator arm. In the current study, patients harboring co-occurring mutations in the ATP-binding pocket and activation loop (*KIT* exons 11 + 13/14 + 17/18) performed similarly irrespective of treatment assignment. However, further investigation is warranted due to the small numbers of patients (11 patients in each treatment arm).

Based on the current findings, a phase 3, randomized, multicenter, open-label study evaluating ripretinib versus sunitinib in patients with advanced GIST previously treated with imatinib who harbor *KIT* exon 11 + 17 and/or 18 mutations (without co-occurring mutations in *KIT* exons 9, 13 or 14) is ongoing (INSIGHT; NCT05734105). In this follow-up phase 3 study, ripretinib was granted breakthrough therapy designation by the US Food and Drug Administration. INSIGHT aims to confirm not only the PFS observed with ripretinib in patients with *KIT* exon 11 + 17/18 mutations (activation loop) from this exploratory analysis, but also the response rate (ORR, 44.4%), which was almost three times the ORR observed with sunitinib in patients with *KIT* exon 11 + 13/14 mutations (ATP-binding pocket; ORR, 15.0%). This finding could be explained by the idea that sunitinib was primarily developed as a potent inhibitor of vascular endothelial growth factor receptor, whereas ripretinib was optimized to inhibit activated KIT (as opposed to competing with ATP binding to the kinase) to decrease unwanted toxicity^38,39^. This difference in response could reflect varied levels of kinase inhibition by ripretinib versus sunitinib against drug-sensitive mutations; however, no on-treatment biopsies were performed to confirm this hypothesis.

Limitations of the current study include the exploratory nature of the analysis; as such, all *P* values reported are nominal and no statistical significance can be claimed or cited in clinical practice. Additionally, there were low patient numbers in some mutational subgroups, making it difficult to interpret some outcomes. There also exists a subset of patients who do not have detectable ctDNA, which does not allow for the personalized treatment approaches proposed in this report. In addition, challenges associated with the technology, such as variables influencing ctDNA stability and sample processing, could contribute to decreased assay sensitivity^24^. Finally, the current study only evaluated imatinib-resistant secondary *KIT* mutations, and further investigation would be necessary to identify any KIT-independent resistance mechanisms.

In conclusion, in this cohort, patients whose ctDNA contained primary *KIT* exon 11 mutations plus secondary mutations restricted to *KIT* exons 17/18 demonstrated greater benefit from ripretinib versus sunitinib, with all the limitations of an exploratory biomarker analysis. In contrast, patients with the same primary mutation (*KIT* exon 11) and secondary mutations restricted to *KIT* exons 13/14 demonstrated greater benefit from sunitinib versus ripretinib. First and foremost, our data suggest that ctDNA analysis may represent a powerful, non-invasive diagnostic tool to identify subgroups of patients with advanced GIST who experienced disease progression on imatinib that may have prolonged clinical benefit from a single TKI therapeutic approach. To this end, ctDNA analysis may broadly determine the heterogeneity of resistance for an individual patient compared with a tissue biopsy, which provides information on only a single lesion. Further investigation of the efficacy of ripretinib as a second-line treatment is required and ongoing in the phase 3 INSIGHT trial (NCT05734105).

## Methods

### Study design

The INTRIGUE trial was conducted in accordance with the Declaration of Helsinki and International Council for Harmonisation Guidelines for Good Clinical Practice. The protocol, protocol amendments and informed consent documents were approved by a central institutional review board (WCG IRB, Puyallup, WA), as well as the institutional review board or ethics committee at each site (Supplementary Information) and by appropriate regulatory authorities. A list of all investigational sites for the INTRIGUE trial was published previously^20^. All patients provided written informed consent at enrollment. Participants were not compensated for participation.

INTRIGUE (NCT03673501) is a randomized, open-label, global, multicenter, phase 3 study comparing efficacy and safety of ripretinib versus sunitinib in patients with advanced GIST who had disease progression on or were intolerant to first-line treatment with imatinib. Patients were stratified by mutational status via tissue biopsy pathology report (*KIT* exon 11, *KIT* exon 9, *KIT*/*PDGFRA* wild-type and other *KIT* mutations (other than exons 9 or 11)/*PDGFRA* mutations) and by imatinib intolerance. Patient sex was self-reported and was not considered in the study design. Patients were randomized (1:1) to receive once-daily ripretinib 150 mg (continuous dosing) or once-daily sunitinib 50 mg (4 weeks on/2 weeks off in 6-week cycles). Crossover was not allowed. The study design and patient disposition were published previously^20^.

### Eligibility criteria

Eligible patients were ≥18 years and had histologically confirmed GIST with one or more measurable lesions by modified Response Evaluation Criteria in Solid Tumors version 1.1 (mRECIST v1.1)^40^ criteria within 21 days before receiving study drug. Eligible patients provided an archival tissue sample and pathology report detailing *KIT/PDGFRA* mutation status by tissue-based PCR or any DNA sequencing analysis, had disease progression with imatinib or demonstrated imatinib intolerance, discontinued imatinib treatment 10 days before the first dose of study drug and had an Eastern Cooperative Oncology Group Performance Status ≤2 with acceptable organ function and bone marrow reserve. Full inclusion/exclusion criteria were published previously^20^.

### ctDNA analysis

In this prespecified exploratory analysis, baseline (cycle 1 day 1) peripheral whole blood was collected in 10-mL Streck cell-free DNA blood collection tubes and shipped to central laboratories for plasma isolation. DNA extraction was performed by Guardant Health and samples were analyzed using Guardant360 (a 74-gene ctDNA NGS–based assay). This assay has a reported 99.6% specificity and 85.0% sensitivity when compared with tissue-based NGS^41^. SNVs and small INDELs can be reported as low as 0.04% and 0.02%, respectively^42^. Mean and median mutant allele frequencies for driver genes *KIT* and *PDGFRA* can be found in Extended Data Table 6.

Patients in the *KIT* exon 11 + 13/14 population have primary mutations in *KIT* exon 11 and secondary resistance mutations only in the KIT ATP-binding pocket (excludes patients with mutations in *KIT* exons 9, 17 and 18). Patients in the *KIT* exon 11 + 17/18 population have primary mutations in *KIT* exon 11 and secondary resistance mutations only in the KIT activation loop (excludes patients with mutations in *KIT* exons 9, 13 and 14). The current exploratory analysis does not include mutational information by tumor biopsy and only provides detailed outcome data for patients with typical secondary *KIT* mutations (ATP-binding pocket and activation loop).

### Outcomes

The primary efficacy endpoint for the INTRIGUE trial was PFS by independent radiologic review (IRR) using mRECIST v1.1; key secondary endpoints were ORR by IRR using mRECIST v1.1 and OS^20^. In the current prespecified exploratory analysis, the baseline mutational landscape was characterized by analyzing the frequency of imatinib-resistant secondary *KIT* mutations in the ATP-binding pocket (exons 13/14) and activation loop (exons 17/18) by position and codon and determining the number of mutations in each patient. Mutational biomarkers that may correlate with treatment response were assessed via PFS, ORR and OS, which were the prespecified, protocol-defined endpoints for the INTRIGUE trial. Safety data are also reported. Data cutoff was 1 September 2021 for all outcomes except OS, which had an updated data cutoff of 1 September 2022.

### Statistical analyses

#### Statistics and reproducibility

In the primary study, patients were randomized (1:1) to receive once-daily ripretinib or once-daily sunitinib. The investigators were not blinded to allocation during experiments and outcome assessment. The endpoints of PFS and ORR were based on IRR and the independent reviewer was blinded to treatment assignment. For this exploratory ctDNA analysis, no statistical method was used to predetermine sample size. The ctDNA assay used all the baseline sample and cannot be reproduced. Of the 374 patient samples received, 12 failed initial quality control review and were not analyzed. No data from the 362 analyzed samples were excluded.

#### Statistical tests

Time-to-event data were summarized using the Kaplan-Meier method with associated two-sided 95% CIs calculated using the Brookmeyer and Crowley method. HRs and *P* values were obtained from the unstratified Cox proportional hazard model and two-sided unstratified log-rank tests, respectively. ORR was analyzed by the chi-square test for association between treatment and ORR; the 95% CI of the RD was calculated using the unstratified Newcombe method. Descriptive statistics were used to summarize safety data. All *P* values reported for this exploratory analysis are nominal. Statistical analyses were done with SAS (version 9.4).

### Reporting summary

Further information on research design is available in the Nature Portfolio Reporting Summary linked to this article.

## Online content

Any methods, additional references, Nature Portfolio reporting summaries, source data, extended data, supplementary information, acknowledgements, peer review information; details of author contributions and competing interests; and statements of data and code availability are available at 10.1038/s41591-023-02734-5.

### Supplementary information

Supplementary Information Supplementary information.

Reporting Summary

## Figures and Tables

**Extended Data Table 1:**
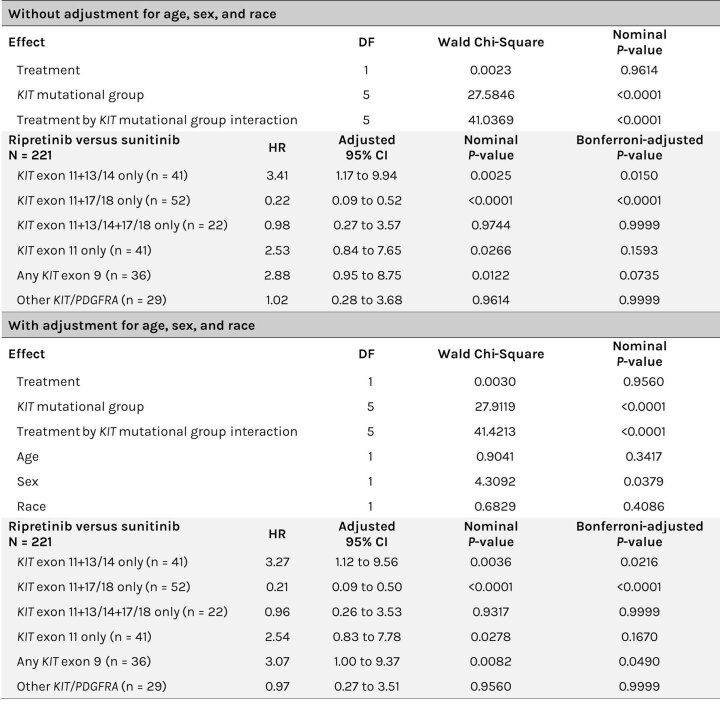
Summary of Cox proportional hazards analysis of PFS examining the interaction of treatment arm and mutational subgroup with or without adjustment for baseline characteristics

The interaction between treatment arm and mutational subgroup was evaluated using the Wald chi-square test; a Cox model with the interaction effect was used and the Bonferroni adjustment was applied. The Bonferroni-adjusted *P*-value was derived by multiplying the nominal *P*-value by the number of comparisons (6). Interaction analysis between treatment arm and mutational subgroup for PFS indicated that the HR values were different across subgroups. The treatment effect was nominally significant in the *KIT* exon 11 + 13/14 and *KIT* exon 11 + 17/18 populations before and after performing the Bonferroni correction. This interaction analysis also showed nominal significance after adjustment for age, sex, and race; females had a significantly lower risk of disease progression or death compared with males, irrespective of treatment or mutation subgroup.

CI, confidence interval; DF, degree of freedom; HR, hazard ratio; PDGFRA, platelet-derived growth factor receptor α; PFS, progression-free survival.

**Extended Data Table 2:**
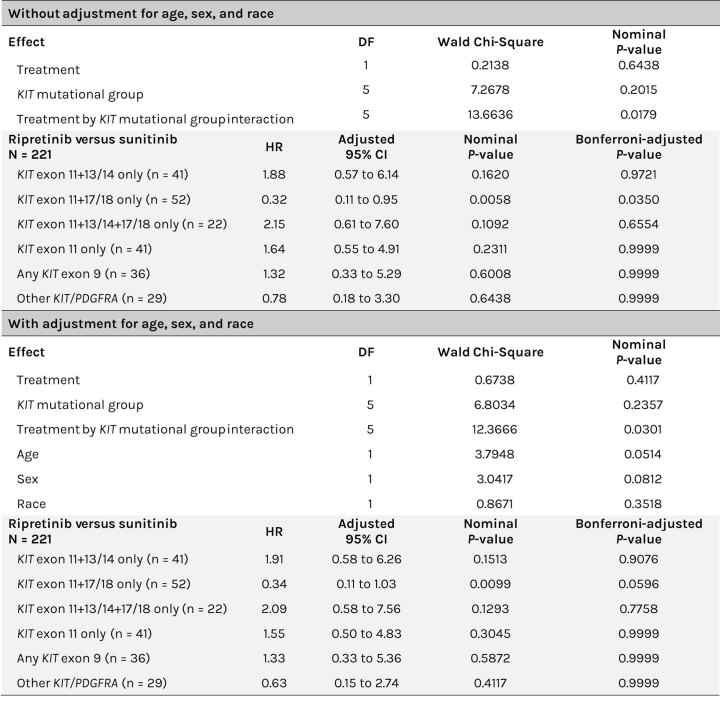
Summary of Cox proportional hazards analysis of OS examining the interaction of treatment arm and mutational subgroup with or without adjustment for baseline characteristics

The interaction between treatment arm and mutational subgroup was evaluated using the Wald chi-square test; a Cox model with the interaction effect was used and the Bonferroni adjustment was applied. The Bonferroni-adjusted *P*-value was derived by multiplying the nominal *P*-value by the number of comparisons (6). Interaction analysis between treatment arm and mutational subgroup for OS indicated that the HR values were different across subgroups. The treatment effect was nominally significant in the *KIT* exon 11 + 17/18 population before and after performing the Bonferroni correction, but not in the *KIT* exon 11 + 13/14 population. This analysis also showed nominal significance after adjustment for age, sex, and race. Females showed a trend towards a lower risk of death compared with males and there was a trend towards a higher risk of death in older patients, irrespective of treatment or mutational subgroup.

CI, confidence interval; DF, degree of freedom; HR, hazard ratio; OS, overall survival; PDGFRA, platelet-derived growth factor receptor α.

**Extended Data Table 3:**
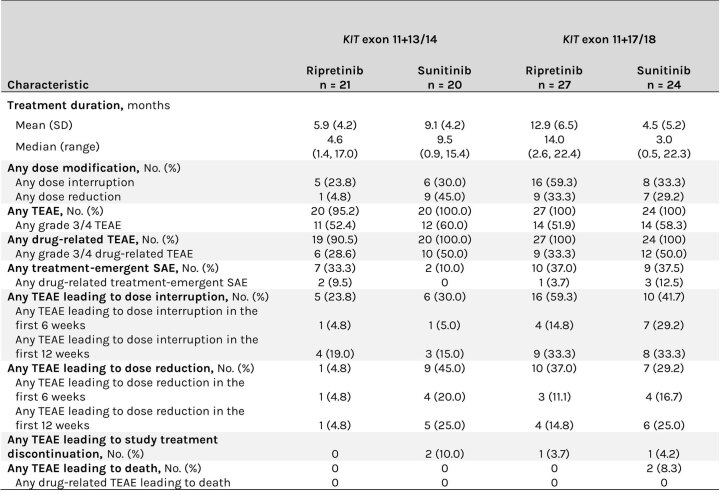
Exposure and AE overview in the *KIT* exon 11 + 13/14 and 11 + 17/18 safety populations

Data cutoff: September 1, 2021.

AEs were graded according to the National Cancer Institute Common Terminology Criteria for Adverse Events version 5.0; AEs were considered treatment emergent if they occurred after administration of the first dose of study drug through 30 days after the last dose of study drug.

AE, adverse event; SAE, serious AE; SD, standard deviation; TEAE, treatment-emergent AE.

**Extended Data Table 4:**
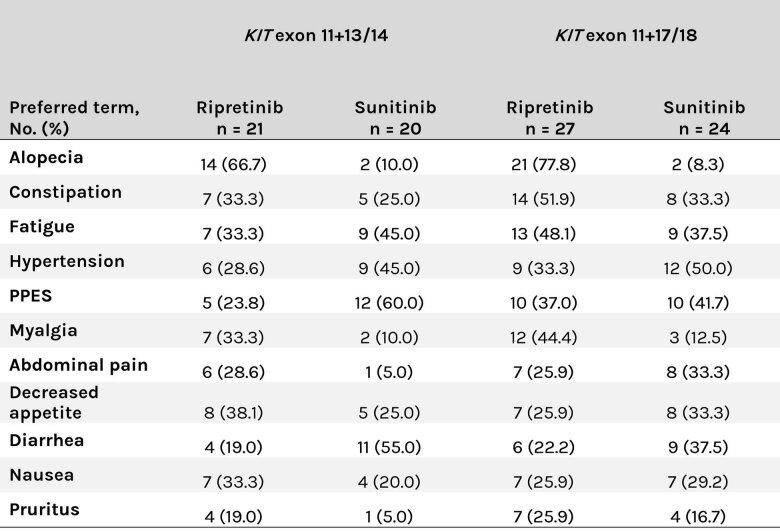
TEAEs in ≥20% of patients in either the *KIT* exon 11 + 13/14 or *KIT* exon 11 + 17/18 safety populations

Data cutoff: September 1, 2021.

AEs were considered treatment emergent if they occurred after administration of the first dose of study drug through 30 days after the last dose of study drug. AEs were coded using MedDRA version 24.0.

AE, adverse event; MedDRA, Medical Dictionary for Regulatory Activities; PPES, palmar-plantar erythrodysesthesia syndrome; TEAE, treatment-emergent AE.

**Extended Data Table 5:**
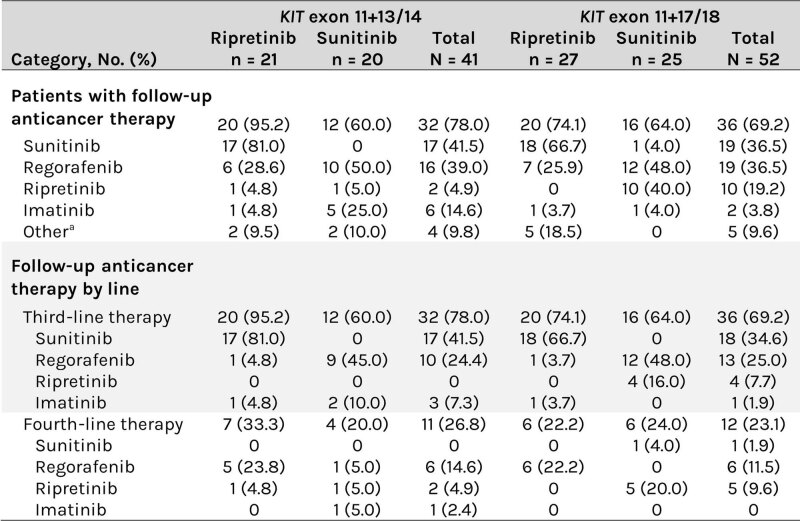
Follow-up anticancer therapies in the *KIT* exon 11 + 17/18 population

Data cutoff: September 1, 2022.

Four patients initiated fifth-line therapy (3 in the ripretinib arm and 1 in the sunitinib arm); 3 patients initiated sixth-line therapy (2 in ripretinib arm and 1 in the sunitinib arm).

^a^Other category includes imatinib + selinexor, avapritinib, unapproved KIT/VEGFR inhibitors (sorafenib, lenvatinib, and pazopanib), antineoplastic agents, investigational drugs, and avelumab.

VEGFR, vascular endothelial growth factor receptor.

**Extended Data Table 6:**
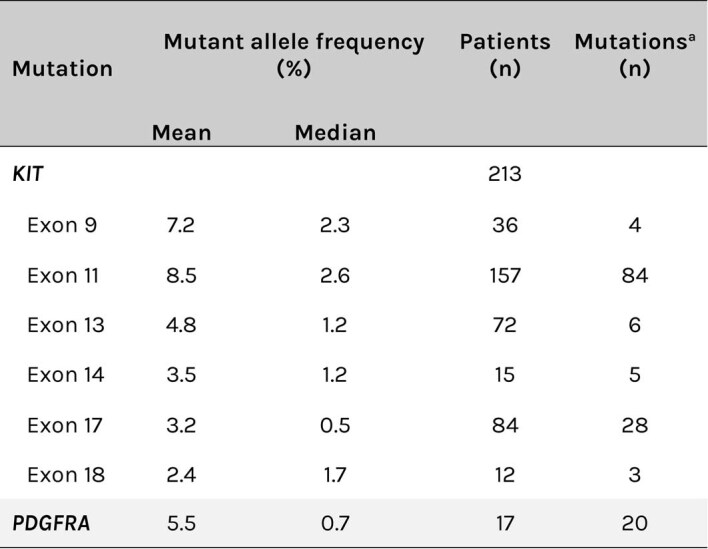
Mutant allele frequencies

Patients can have >1 mutation.

^a^Represents the number of unique mutations.

PDGFRA, platelet-derived growth factor receptor α.

## Data Availability

The redacted study protocol for the INTRIGUE trial was previously published and can be accessed here: https://ascopubs.org/doi/suppl/10.1200/JCO.22.00294/suppl_file/protocol_JCO.22.00294.pdf. The ctDNA dataset contains person-sensitive data and is not broadly available due to privacy laws. Qualified scientific and medical researchers can make requests for individual participant data that underlie the results reported in this article, after de-identification, at info@deciphera.com. Proposals for data will be evaluated and approved by Deciphera in its sole discretion. All approved researchers must sign a data access agreement before accessing the data. Data will be available as soon as possible but no later than within 1 year of the acceptance of the article for publication and for 3 years after article publication. Deciphera will not share data from identified participants or a data dictionary.

## Extended data

are available for this paper at 10.1038/s41591-023-02734-5.
