# Supplementary material for: Ripretinib versus sunitinib in gastrointestinal stromal tumor: ctDNA biomarker analysis of the phase 3 INTRIGUE trial
Source: Nat Med. 2024 Jan 5;30(2):498–506. doi: 10.1038/s41591-023-02734-5 (PMC10878977; doi:10.1038/s41591-023-02734-5)
Supplement: Supplementary file 1 — Supplementary information. [file 41591_2023_2734_MOESM1_ESM.pdf]

nature medicine

Article https://doi.org/10.1038/s41591-023-02734-5

Ripretinib versus sunitinib in gastrointestinal stromal tumor: ctDNA biomarker analysis of the phase 3 INTRIGUE trial

In the format provided by the authors and unedited

Supplementary Information

The protocol, protocol amendments, and informed consent documents were approved by the institutional review board (IRB) or ethics committee at each site.

In the US, the following centers participated:

- Office for Human Research Studies, Dana-Farber Cancer Institute, Boston, MA
- OHSU Research Integrity Office, Oregon Health & Science University (OHSU), Portland, OR
- Stanford IRB Research Compliance Office, Stanford Hospital and Clinics, Stanford, CA
- University of Chicago IRB, University of Chicago Medical Center, Chicago, IL
- UCLA IRB, UCLA Hematology Oncology - Main Site, Los Angeles, CA
- MD Anderson Cancer Center IRB, U T MD Anderson Cancer Center Investigational Pharmacy Services Unit 376, Houston, TX
- Mayo Clinic IRB, Mayo Clinic in Florida, Jacksonville, FL
- University of Minnesota Institutional Review Board, University of Minnesota Hospital, Minneapolis, MN
- Mayo Clinic IRB, Mayo Clinic, Rochester, MN
- Northwestern University Institutional Review Board, Northwestern Center for Clinical Research, Chicago, IL
- Duke University Health System Institutional Review Board, Duke University Medical Center, Durham, NC
- University of Miami Human Subject Research Office IRB, University of Miami Miller School of Medicine, Miami, FL
- UCSD Human Research Protection Program, University of California San Diego Medical Center, San Diego, CA

- Biomedical Research Alliance of New York, LLC (BRANY), Montefiore Medical Center (MMC), Bronx, NY
- Vanderbilt University Medical Center IRB, Vanderbilt University Medical Center, Nashville, TN
- Johns Hopkins University IRB, Johns Hopkins University School Of Medicine, Baltimore, MD
- UTMC BioMedical IRB, University of Toledo, Toledo, OH
- Washington University in St. Louis IRB, Washington University, School of Medicine, St. Louis, MO
- Henry Ford Health Systems IRB, Henry Ford Hospital, Detroit, MI
- Medical College of Wisconsin/Froedtert Hospital IRB, Medical College of Wisconsin, Inc., Milwaukee, WI
- University of Iowa-IRB, University of Iowa Hospital and Clinics, Iowa City, IA
- Biomedical Research Alliance of New York, LLC (BRANY), The Monter Cancer Center, New Hyde Park, NY
- US Oncology, Inc. IRB, Baptist Health Medical Group Oncology, LLC, Miami, FL
- US Oncology, Inc. IRB, Rocky Mountain Cancer Centers, LLP, Denver, CO
- US Oncology, Inc. IRB, Baylor Scott & White Research Institute, Dallas, TX
- Mayo Clinic IRB, Mayo Clinic Arizona, Phoenix, AZ
- Cleveland Clinic IRB, Cleveland Clinic, Cleveland, OH
- Advarra, H. Lee Moffitt Cancer Center and Research Institute, Inc., Tampa, FL

Outside the US, the following centers, IRBs, and ethics committees participated:

- Comite de Etica Instituto Medico Especializado Alexander Fleming, Argentina
- COEIS-Consejo de Evaluación Ética de la Investigación en Salud, Argentina

- Comite de Etica del Sanatorio Allende, Argentina
- Alfred Hospital Ethic Committee, Australia
- South Eastern Sydney LHD Human Research Ethics Committee (RGO), Australia
- Metro South Health Service District Human Research Ethics Committee (RGO), Australia
- Bellberry Human Research Ethics Committees (Non Lead Sites), Australia
- Bellberry Human Research Ethics Committees, Australia
- Ethische Commissie Onderzoek UZ/KU Leuven, Belgium
- University Health Network Research Ethics Board, Canada
- Health Research Ethics Board of Alberta - Cancer Committee (HREBA-CC), Canada
- Maisonneuve-Rosemont hospital REB, Canada
- Ontario Cancer Research Ethics Board, Canada
- Nova Scotia Health Authority Research Ethics Board, Canada
- Comité Ético Científico de la Facultad de Medicina de la Pontificia Universidad Católica de Chile, Chile
- Comité de Protection des Personnes Ile de France II, France
- Ethikkommission an der Technischen Universität Dresden-LEC, Germany
- Ethik-Kommission der Medizinischen Fakultaet der Universitaet Duisburg-Essen, Germany
- Egeszsegugyi Tudomanyos Tanacs Klinikai Farmakologiai Etikai Bizottsaga, Hungary
- Tel Aviv Sourasky, Israel
- Rambam Health Care Campus Ethics Committee, Israel
- Comitato Etico Indipendente della Fondazione IRCCS Istituto Nazionale dei Tumori di Milano, Italy

- Comitato Etico Territoriale Lombardia 4, Italy
- Comitato Etico Università Campus Bio-Medico di Roma, Italy
- Comitato Etico IRCCS Istituto Oncologico Veneto di Padova, Italy
- Comitato Etico IRCCS di Candiolo, Italy
- Comitato Etico Indipendente di Area Vasta Emilia Centro (CE-AVEC), Italy
- Comitato Etico Palermo 1, Italy
- IRB of Asan Medical Center, Republic of Korea
- IRB of Ajou University Hospital, Republic of Korea
- IRB of Samsung Medical Center, Republic of Korea
- IRB of Seoul National University Hospital, Republic of Korea
- Ethics Committee Leiden, Netherlands
- REK Sør-øst, Norway
- Komisja Bioetyczna przy Centrum Onkologii, Poland
- SingHealth Centralised Institutional Review Board, Singapore
- Hospital Universitari Vall d'Hebron, Spain
- Regionala etikprövningsnämnden i Stockholm, Sweden
- Commission cantonale d'éthique de la recherche sur l'être humain (CER-VD), Switzerland
- China Medical University Hospital, Institutional Review Board, Taiwan
- National Cheng Kung University Hospital, Institutional Review Board, Taiwan
- Chang Gung Medical Foundation, Institutional Review Board, Taiwan
- Taipei Veterans General Hospital, Institutional Review Board, Taiwan
- NRES Committee London - City & East, United Kingdom
- R&D - The Royal Marsden NHS Foundation Trust, United Kingdom

- R&D The Leeds Teaching Hospitals NHS Trust, United Kingdom
- R&D UCLH NHS Foundation Trust, United Kingdom
