# Supplementary material for: Ripretinib versus sunitinib in gastrointestinal stromal tumor: ctDNA biomarker analysis of the phase 3 INTRIGUE trial
Source: Nat Med. 2024 Jan 5;30(2):498–506. doi: 10.1038/s41591-023-02734-5 (PMC10878977; doi:10.1038/s41591-023-02734-5)
Supplement: Supplementary file 2 — Reporting Summary [file 41591_2023_2734_MOESM2_ESM.pdf]

nature portfolio

Corresponding author(s): Sebastian Bauer

Last updated by author(s): Oct 30, 2023

Reporting Summary

Nature Portfolio wishes to improve the reproducibility of the work that we publish. This form provides structure for consistency and transparency in reporting. For further information on Nature Portfolio policies, see our Editorial Policies and the Editorial Policy Checklist.

Statistics

For all statistical analyses, confirm that the following items are present in the figure legend, table legend, main text, or Methods section.

n/a	Confirmed	
☐	☒	The exact sample size (*n*) for each experimental group/condition, given as a discrete number and unit of measurement
☐	☒	A statement on whether measurements were taken from distinct samples or whether the same sample was measured repeatedly
☐	☒	The statistical test(s) used AND whether they are one- or two-sided<br>*Only common tests should be described solely by name; describe more complex techniques in the Methods section.*
☒	☐	A description of all covariates tested
☐	☒	A description of any assumptions or corrections, such as tests of normality and adjustment for multiple comparisons
☐	☒	A full description of the statistical parameters including central tendency (e.g. means) or other basic estimates (e.g. regression coefficient) AND variation (e.g. standard deviation) or associated estimates of uncertainty (e.g. confidence intervals)
☐	☒	For null hypothesis testing, the test statistic (e.g. *F*, *t*, *r*) with confidence intervals, effect sizes, degrees of freedom and *P* value noted<br>*Give P values as exact values whenever suitable.*
☒	☐	For Bayesian analysis, information on the choice of priors and Markov chain Monte Carlo settings
☐	☒	For hierarchical and complex designs, identification of the appropriate level for tests and full reporting of outcomes
☒	☐	Estimates of effect sizes (e.g. Cohen's *d*, Pearson's *r*), indicating how they were calculated

Our web collection on statistics for biologists contains articles on many of the points above.

Software and code

Policy information about availability of computer code

Data collection	No specific software was used for data collection; the collected data were entered using the IBM (now Zelta) electronic data collection system; the mint Lesion™ application was used to collect tumor response data from the independent radiologic review.
Data analysis	Statistical analyses were done with SAS (version 9.4; Cary, NC)

For manuscripts utilizing custom algorithms or software that are central to the research but not yet described in published literature, software must be made available to editors and reviewers. We strongly encourage code deposition in a community repository (e.g. GitHub). See the Nature Portfolio guidelines for submitting code & software for further information.

Data

Policy information about availability of data

All manuscripts must include a data availability statement. This statement should provide the following information, where applicable:

- Accession codes, unique identifiers, or web links for publicly available datasets
- A description of any restrictions on data availability
- For clinical datasets or third party data, please ensure that the statement adheres to our policy

The redacted study protocol for the INTRIGUE trial was previously published and can be accessed here: https://ascopubs.org/doi/suppl/10.1200/JCO.22.00294/suppl_file/protocol_JCO.22.00294.pdf. The ctDNA dataset contains person-sensitive data and is not broadly available due to privacy laws. Qualified scientific and medical researchers can make requests for individual participant data that underlie the results reported in this article, after de-identification, at

info@deciphera.com. Proposals for data will be evaluated and approved by Deciphera in its sole discretion. All approved researchers must sign a data access agreement before accessing the data. Data will be available as soon as possible but no later than within 1 year of the acceptance of the article for publication, and for 3 years after article publication. Deciphera will not share data from identified participants or a data dictionary.

Research involving human participants, their data, or biological material

Policy information about studies with human participants or human data. See also policy information about sex, gender (identity/presentation), and sexual orientation and race, ethnicity and racism.

Reporting on sex and gender

Patient sex was self-reported and not considered in the INTRIGUE study design. In the entire INTRIGUE study, 62% of patients were male and 38% were female. In the KIT exon 11+13/14 population, 51% of patients were male and 49% were female. In the KIT exon 11+17/18 population, 73% were male and 27% were female.

Reporting on race, ethnicity, or other socially relevant groupings

Patient demographics are reported in Table 1 of the manuscript. Race was self-reported. Most patients in the INTRIGUE study were white and from North America or Europe. In the KIT exon 11+13/14 population, 27 (66%) of patients were white, 3 (7%) were Asian, 5 (12%) were black, and 6 (15%) did not report their race; in the KIT exon 11+17/18 population, 37 patients (71%) were white, 6 (12%) were Asian, 4 (8%) were black, 1 (2%) was Native Hawaiian or other Pacific Islander, and 4 (8%) did not report their race.

Population characteristics

This exploratory analysis was based on the presence of secondary resistance mutations. The breakdown of mutations detected by baseline ctDNA analysis is provided in Figure 1 of the manuscript (disposition flowchart). Initial results identified two diametrically opposed populations: patients with primary KIT exon 11 mutations with imatinib-resistant mutations exclusively in exons 13/14 (41/362 [11%]) and in patients with primary KIT exon 11 mutations with imatinib-resistant mutations exclusively in exons 17/18 (52/362 [14%]). Population characteristics for these two focused populations can be found in Table 1 of the manuscript. In the KIT exon 11+13/14 population, the median age was 59 years; in the KIT exon 11 +17/18 population, the median age was 60 years. Also reported in this table are relevant clinical characteristics such as primary tumor site, Eastern Cooperative Oncology Group Performance status, imatinib intolerance, sum of the longest diameters of target lesions, and duration of imatinib therapy.

Recruitment

Patients were recruited based on diagnosis and treatment plan. No self selection bias was present.

Ethics oversight

The INTRIGUE trial was conducted in accordance with the Declaration of Helsinki and International Council for Harmonisation Guidelines for Good Clinical Practice. The protocol, protocol amendments, and informed consent documents were approved by a central institutional review board (WCG IRB, Puyallup, WA), as well as the institutional review board or ethics committee at each site, and by appropriate regulatory authorities. A list of all investigational sites for the INTRIGUE trial was published previously. All patients provided written informed consent at enrollment. Participants were not compensated for participation.

Note that full information on the approval of the study protocol must also be provided in the manuscript.

Field-specific reporting

Please select the one below that is the best fit for your research. If you are not sure, read the appropriate sections before making your selection.

☒ Life sciences ☐ Behavioural & social sciences ☐ Ecological, evolutionary & environmental sciences

For a reference copy of the document with all sections, see nature.com/documents/nr-reporting-summary-flat.pdf

Life sciences study design

All studies must disclose on these points even when the disclosure is negative.

Sample size

Because this was an exploratory analysis, no sample size calculation was performed. Sample sizes were determined based on the KIT mutational status. We note here and in the manuscript that due to the exploratory nature of this analysis, all reported P-values are nominal and no statistical significance can be claimed.

Data exclusions

Of 453 patients in the INTRIGUE study, samples were available for 374 patients. Of those 374 samples, 12 were excluded because the sample failed quality control checks. The total ctDNA analysis population included 362 patients.

Replication

Data cannot be replicated because additional samples are not available.

Randomization

Not relevant to this manuscript because this was an exploratory analysis. In the overall INTRIGUE trial, patients were randomly allocated to treatment conditions. Here, we report an excerpt from the primary manuscript (Bauer et al. J Clin Oncol. 2022): "Patients were stratified by mutational status (KIT exon 11, KIT exon 9, KIT/PDGFRA wild-type [WT], and other KIT [other than exon 9 or exon 11]/PDGFRA mutations) and imatinib intolerance and subsequently randomly assigned (1:1) to receive once-daily ripretinib 150 mg (continuous dosing) or once-daily sunitinib 50 mg, 4 weeks on/2 weeks off (4/2) in 6-week cycles. Crossover was not allowed." Please note that the mutation status used for stratification was based on local pathology report and not baseline ctDNA.

Blinding

INTRIGUE is an open-label study. However, the endpoints of progression-free survival and objective response rate were based on independent radiologic review and the independent reviewer was blinded to treatment assignment.

Reporting for specific materials, systems and methods

We require information from authors about some types of materials, experimental systems and methods used in many studies. Here, indicate whether each material, system or method listed is relevant to your study. If you are not sure if a list item applies to your research, read the appropriate section before selecting a response.

Materials & experimental systems

n/a	Involved in the study
☒	☐ Antibodies
☒	☐ Eukaryotic cell lines
☒	☐ Palaeontology and archaeology
☒	☐ Animals and other organisms
☐	☒ Clinical data
☒	☐ Dual use research of concern
☒	☐ Plants

Methods

n/a	Involved in the study
☒	☐ ChIP-seq
☒	☐ Flow cytometry
☒	☐ MRI-based neuroimaging

Clinical data

Policy information about clinical studies

All manuscripts should comply with the ICMJE guidelines for publication of clinical research and a completed CONSORT checklist must be included with all submissions.

Clinical trial registration: Clinicaltrials.gov identifier: NCT03673501

Study protocol: The study protocol was published previously: https://ascopubs.org/doi/suppl/10.1200/JCO.22.00294/suppl_file/protocol_JCO.22.00294.pdf

Data collection: INTRIGUE was active at 122 sites in 22 countries. These sites were typically cancer centers affiliated with academic hospitals. The full list of sites was published with the primary manuscript (Bauer et al. J Clin Oncol. 2022; Table A1). Patient recruitment occurred from February 8, 2019 to December 22, 2020. Data for this manuscript was collected from February 8, 2019 to September 1, 2021 (for progression-free survival, overall response rate, and safety) or September 1, 2022 (for overall survival).

Outcomes: The primary endpoint of the INTRIGUE study was progression-free survival (PFS) based on independent radiologic review (IRR) using mRECIST v1.1. The independent reviewer was blinded to treatment assignment. PFS was defined as the time interval between the date of randomization and the earliest documented evidence of the first disease progression based on IRR or death due to any cause, whichever occurred first. Key secondary endpoints included objective response rate (ORR), overall survival (OS), and safety. ORR was defined as the proportion of patients who achieved confirmed complete response or partial response based on IRR per mRECIST v1.1. OS was defined as time from date of randomization until death. All participants were followed until withdrawal of consent or death from any cause to collect long-term survival data. Adverse events were considered treatment-emergent if they occurred after the first dose of the study drug through 30 days after the last dose of study drug or the day before the start of subsequent new anticancer drug therapy, whichever occurred first. Drug-related events reported after 30 days after the last dose of study drug were also considered treatment-emergent.

Plants

Seed stocks: *Report on the source of all seed stocks or other plant material used. If applicable, state the seed stock centre and catalogue number. If plant specimens were collected from the field, describe the collection location, date and sampling procedures.*

Novel plant genotypes: *Describe the methods by which all novel plant genotypes were produced. This includes those generated by transgenic approaches, gene editing, chemical/radiation-based mutagenesis and hybridization. For transgenic lines, describe the transformation method, the number of independent lines analyzed and the generation upon which experiments were performed. For gene-edited lines, describe the editor used, the endogenous sequence targeted for editing, the targeting guide RNA sequence (if applicable) and how the editor was applied.*

Authentication: *Describe any authentication procedures for each seed stock used or novel genotype generated. Describe any experiments used to assess the effect of a mutation and, where applicable, how potential secondary effects (e.g. second site T-DNA insertions, mosiacism, off-target gene editing) were examined.*
